# Lymphocyte Transformation Test (LTT) in Allergy to Benznidazole: A Promising Approach

**DOI:** 10.3389/fphar.2019.00469

**Published:** 2019-05-21

**Authors:** M. Andreína Marques-Mejías, Rosario Cabañas, Elena Ramírez, Javier Domínguez-Ortega, Ana Fiandor, Elena Trigo, Santiago Quirce, Teresa Bellón

**Affiliations:** ^1^Department of Allergy, La Paz University Hospital, Madrid, Spain; ^2^PIELenRed Consortium, Madrid, Spain; ^3^Hospital La Paz Institute for Health Research (IdiPaz), Madrid, Spain; ^4^Pharmacology Department, La Paz University Hospital, Madrid, Spain; ^5^Tropical Medicine and Travel Health Unit, Department of Internal Medicine, University Hospital La Paz, Madrid, Spain

**Keywords:** benznidazole allergy, lymphocyte transformation test, drug allergy, delayed hypersensitivity, benznidazole, benznidazole sensitivity

## Abstract

Benznidazole (Bzn) from the nitroimidazole family and nifurtimox from nitrofurans family, are drugs used as first and second line treatment for acute and chronic phases of Chagas disease (CD). Even though skin reactions are frequent, confirmed allergy to Bzn is rare, and there are few cases reported in the literature. Since CD treatment is very restrained, the possibility of cross-reactivity between members of the same and other pharmacological families highlights the importance of an adequate diagnosis that allows alternative treatments in CD and other diseases. We report a series of 31 patients (69% women) referred to our Allergy unit with suspected hypersensitivity to Bzn, twenty three of them with mild reactions and eight of them with severe reactions. LTT with Bzn was performed in 31 patients and in 8 negative controls. LTT was also performed in 25 and 20 of these patients with nifurtimox and Mtn, respectively. Twenty-one out of thirty-one patients were Bzn prick tested, and all were negative. We obtained 2/19 positive results on patch tests to Bzn. LTT with Bzn was positive in 22/31 patients (Sensitivity 75.9% and specificity 100%). The test was considered positive with a stimulation index ≥2. There was a positive result in 7/25 patients for nifurtimox and in 7/20 patients with Mtn. After negative LTT and skin tests, oral provocation was performed in 4/9 patients, all negative. LTT is a safe test that seems to be more useful than skin tests (prick and patch test), particularly in severe reactions, in confirming delayed hypersensitivity to Bzn and detecting cross reactivity with other imidazoles such as Mtn and reactivity to other drugs like nifurtimox. Tests for these drugs need to be included in the workup of patients with hypersensitivity to Bzn in case they are needed as an alternative treatment for CD or to treat other frequent infectious diseases.

## Introduction

Benznidazole (Bzn) from the nitroimidazole family and nifurtimox from nitrofurans family, are drugs used as first and second line treatment for acute and chronic phases of Chagas disease (CD) (Noguerado-Mellado et al., 2017). This treatment has not been modified for more than 100 years after the discovery of the disease because to date, there are no other drugs as effective (Pérez-Molina et al., 2013). Metronidazol (Mtn) also belongs to the nitroimidazole family. It is not used to treat CD, but to treat other infectious diseases caused by parasites and anaerobic bacteria (Noguerado-Mellado et al., 2017).

Even though skin reactions are frequent (Carrilero et al., 2011), confirmed allergy to Bzn is rare and there are few cases reported in the literature (Álava-Cruz et al., 2014; González-Ramos et al., 2016; Noguerado-Mellado et al., 2017; Moreno-Escobosa and Cruz- Granados, 2018). Since CD treatment is very restrained, the possibility of cross-reactivity between members of the same and other pharmacological families highlights the importance of an adequate diagnosis that allows for alternative treatments in CD and other diseases.

The lymphocyte transformation test (LTT) relies on the ability of drug-specific memory T cells to proliferate once they have been stimulated by an antigen (Cabañas et al., 2018). Like other *in vitro* tests, it is safer than *in vivo* tests available (Lochmatter et al., 2009). To the best of our knowledge, this is the biggest series reported to this date of hypersensitivity reactions to Bzn, including both mild and severe clinical manifestations. It is also the first one in which an *in vitro* diagnostic test was used, such as LTT. Thus far, there was only record of cross-reactivity between Bzn and metronidazole (Prieto et al., 2005; Pérez-Molina et al., 2013; Noguerado-Mellado et al., 2017).

## Materials and Methods

We report a series of 31 patients (69% women) referred to our Allergy unit with suspected hypersensitivity to Bzn, twenty-three of them with mild reactions such as exanthema/urticaria and angioedema and eight of them with severe reactions like drug reaction with eosinophilia and systemic symptoms (DRESS), Stevens Johnson syndrome (SJS)/ toxic epidermal necrolysis (TEN), or acute generalized exanthematous pustulosis (AGEP), (Table 1). None of the patients had any history of reaction to other drugs. All the patients included in the sample were originally from Bolivia and El Salvador, but currently living in Madrid, Spain. LTT with Bzn was performed in 31 patients and in 8 controls. LTT was also performed in 25 and 20 of these patients with nifurtimox and Mtn, respectively. LTT methodology is detailed elsewhere (Pichler and Tilch, 2004). Briefly, mononuclear cells from peripheral blood were stimulated with increasing concentrations of Bzn over 6 days in the presence of 5% autologous serum, and the proliferation was evaluated through the incorporation of ^3^H-thyminide to DNA. Positive control cultures were performed in the presence of phytohaemagglutinin (Sigma). The stimulation index (SI) was calculated as the ratio of ^3^H incorporated by drug-stimulated cultures and basal ^3^H incorporation by unstimulated cells (Cabañas et al., 2018). As the standard criteria, SI ≥ 2 in at least one concentration was considered positive. LTT was performed after recovery at least 1 month after steroid treatment was stopped. Bzn and nifurtimox were purchased from Sigma-Aldrich and stock solutions were prepared in DMSO. Dilutions were freshly prepared in RPMI cell culture medium just before use. For Mtn testing, crushed pills were dissolved in RPMI cell culture medium. The resulting solution was centrifuged and sterile filtered before use.

**Table 1:** Diagnosis and latency time.

Patients	Latency time (days)	Diagnosis
1	61	urticaria
2	60	macular exantema
3	10	exantema
4	28	papular exantema
5	10	urticaria
6	7	urticaria
7	5	urticaria
8	83	urticaria
9	10	pruritus
10	7	urticaria
11	30	urticaria
2	7	exantema
3	11	urticaria
4	10	papular exantema
5	2	exantema, facial angioedema
6	11	urticaria, facial angioedema
7	39	urticaria
8	10	urticaria
9	11	urticaria
20	45	pruritus
21	5	exantema
22	9	urticaria, angioedema facial
23	9	exantema
24	21	DRESS/TEN
25	1	AGEP
26	10	DRESS
27	8	DRESS
28	10	DRESS
29	5	DRESS
30	25	DRESS/SJS
31	20	DRESS

*DRESS, drug reaction with eosinophilia and systemic symptoms; SJS, Steven Johnsons syndrome; AGEP, acute generalized exanthematous pustulosis, TEN, toxic epidermal necrolysis.*

## Results

The mean age of the sample was 41 year (SD ± 9) for mild reactions and 38 year (SD ± 9) for severe reactions. Every patient received treatment with 250–300 mg of Bzn per day. The latency time between the beginning of treatment and the appearance of symptoms ranged from 2 to 61 days (mean = 21.3) for mild reactions and between 1 and 25 days (mean = 13) for severe reactions (*p* < 0.05) (Table 1).

Twenty one out of thirty-one patients were Bzn prick tested, and all were negative. We obtained 2/19 positive results on patch tests to Bzn. LTT with Bzn was performed in 31 patients with non-immediate reactions to Bzn, including those with severe reactions (22/31 positive). The test was considered positive with a stimulation index ≥2. There was a positive result in 7/25 patients for nifurtimox and in 7/20 patients with Mtn. Five of them were both reactive to nifurtimox and Mtn in addition to Bzn. After negative LTT and skin tests, oral provocation was performed in 4/9 patients, and all were negative (Table 2). A total of eight tolerant patients to Bzn were also studied with LTT assay, showing specificity of 85.7%.

**Table 2:** Allergy workup.

	Prick-test		Patch test		LTT			
Patients	BZN	Nifurtimox	BZN	Nifurtimox	BZN	Nifurtimox	MTN	ASPS
1	–	NP	–	NP	+	+	–	7
2	–	NP	–	–	+	+	+	7
3	–	–	–	–	+	–	–	7
4	NP	NP	–	NP	+	+	+	7
5	–	NP	–	NP	–	–	–	7
6	–	NP	NP	NP	+	+	+	6
7	–	NP	–	NP	–	–	NP	10
8	–	NP	NP	NP	+	+	–	7
9	–	NP	NP	NP	+	–	–	7
10	–	NP	–	–	+	–	+	6
11	–	–	–	–	+	–	+	7
12	–	NP	–	NP	+	–	NP	7
13	–	NP	NP	NP	+	–	NP	6
14	NP	NP	–	NP	+	–	–	6
15	–	NP	–	NP	–	–	–	6
16	–	NP	–	NP	–	–	–	3
17	–	NP	+	NP	–	NP	NP	11
18	NP	NP	–	NP	–	NP	NP	6
19	–	NP	–	NP	–	–	NP	6
20	–	NP	–	NP	+	+	+	6
21	–	–	–	–	–	–	–	7
22	NP	NP	NP	NP	–	–	–	7
23	–		–	–	+	–	–	7
24	–	NP	NP	NP	+	–	–	7
25	NP	NP	NP	NP	+	NP	NP	7
26	NP	NP	NP	NP	+	NP	NP	7
27	NP	NP	NP	NP	+	NP	NP	7
28	NP	NP	NP	NP	+	–	–	7
29	NP	–	NP	NP	+	–	NP	7
30	–	–	+	–	+	+	+	7
31	NP	NP	NP	NP	+	NP	NP	9

*ASPS, Algorithm of the Spanish Pharmacovigilance System for drug causality assessment; Bzn, benznidazole; Mtn, metronidazole; LTT, lymphocyte transformation test; NP, not performed.*

To assess the sensitivity of LTT with Bzn, we used the ASPS (Algorithm of the Spanish Pharmacovigilance System for drug causality assessment) which has been proven as a suitable approach for evaluation of diagnostic capacity of LTT (Cabañas et al., 2018). We considered ASPS ≥ 4 as the standard for a correct diagnosis, finding an overall sensitivity of 75.9 and 100% specificity. For mild reactions, a sensitivity of 66.7% and specificity of 100% was obtained. As for severe reactions, there was a perfect correlation between LTT and ASPS (sensitivity of 100% and specificity of 100%).

## Discussion

Despite some published data that sustained that patients with hypersensitivity reactions to Bzn could tolerate nifurtimox (Pérez-Molina et al., 2013), we found that 33% of the patients that had positive LTT with Bzn also had a positive LTT with nifurtimox. This led us to propose that previous allergy tests should be performed once a patient develops a hypersensitivity reaction to Bzn before treating him/her with nifurtimox. Five out of twenty-one patients (23.8%) were also reactive with both nifurtimox and Mtn in addition to Bzn. Our results regarding Mtn match the percentage of cross-reactivity reported in previous series (33.3%) (Noguerado-Mellado et al., 2017).

Benznidazole and Mtn both belong to nitroimidazole family and share structural features. However, in patients with positive LTT to both Bzn and nifurtimox, it is unlikely to be related to “cross-reactivity” due to lack of structural resemblance. Cases with reactivities to drugs without any structural similarity have been published (Chou et al., 2015).

It has been suggested that cross-reactivity can appear with memory T cells previously expanded in response to other pathogens, a phenomenon called heterologous immunity (White et al., 2015). As the patients had not been previously exposed to nifurtimox, it is tempting to speculate that this mechanism could explain our results (and the high frequency to adverse reactions to Bzn and nifurtimox). Another alternative mechanism, from a pharmacokinetic point of view, could be the formation of free radicals or other metabolites that could promote these types of reactions (Ju and Uetrecht, 2002) but further studies need to be performed in this regard.

A total of eight tolerant patients to Bzn were also studied with LTT assay, showing specificity of 85.7%, similarly to previous results (Nyfeler and Pichler, 1997); nonetheless, a lower specificity of LTT to nifurtimox (leading to false-positive results) is possible. Further research is needed to explore the sensitivity and specificity of LTT to nifurtimox in tolerant but sensitized donors.

## Conclusion

In conclusion, LTT is a safe test that seems to be more useful than skin tests (prick and patch test), particularly in severe reactions, in confirming delayed hypersensitivity to Bzn and detecting cross reactivity with other imidazoles such as Mtn and possibly, reactivity to other drugs like nifurtimox. Tests for these drugs need to be included in the workup of patients with hypersensitivity to Bzn, in case they are needed as an alternative treatment for CD or to treat other frequent infectious diseases.

## Ethics Statement

The study was approved by the local Ethics Committee (Code PI-1674). All subjects gave written informed consent in accordance with the Declaration of Helsinki.

## Author Contributions

MM-M was responsible for data collection, redaction of the manuscript, and for the Algorithm of the Spanish Pharmacovigilance System for drug causality assessment. RC contributed towards data collection, redaction, and correction of the manuscript. ER contributed towards the Algorithm of the Spanish Pharmacovigilance System for drug causality assessment, and correction of the manuscript. JD-O contributed towards the compilation of patients and correction of the manuscript. ET and AF both contributed towards the compilation of patients. SQ contributed towards the correction of the manuscript. TB was responsible for the overall supervision of the manuscript and for conducting LTTs on every patient.

## Conflict of Interest Statement

The authors declare that the research was conducted in the absence of any commercial or financial relationships that could be construed as a potential conflict of interest.
